# Two new species and one new record of *Bolbitius* (*Bolbitiaceae*, *Agaricales*) from northeast China

**DOI:** 10.3897/mycokeys.140.203173

**Published:** 2026-09-23

**Authors:** Hong Cheng, Tolgor Bau

**Affiliations:** 1 College of Mycology, Jilin Agricultural University, Changchun 130118, Jilin, China College of Mycology, Jilin Agricultural University Changchun China; 2 Key Laboratory of Edible Fungal Resources and Utilization (North), Ministry of Agriculture and Rural Affairs, Jilin Agricultural University, Changchun 130118, Jilin, China Key Laboratory of Edible Fungal Resources and Utilization (North), Ministry of Agriculture and Rural Affairs, Jilin Agricultural University Changchun China

**Keywords:** *
Bolbitius
*, multi-gene fragments, morphology, new taxa, phylogeny

## Abstract

A taxonomic study of the genus *Bolbitius* from northeast China was conducted, based on morphological observations and molecular phylogenetic analyses. Phylogenetic relationships were reconstructed, based on a combined multi-locus dataset comprising the internal transcribed spacer (ITS), nuclear large subunit (nrLSU) and translation elongation factor 1-α (*tef1-α*) sequences. As a result, two new species are described and illustrated: *Bolbitius planoflavus*, characterised by its distinctly applanate, pale yellow pileus when mature and the presence of occasional 2-spored basidia; and *Bolbitius fuscopapillatus*, distinguished by its violet-brown papillate umbo and purplish-white margin bearing faint veil remnants. Additionally, *Bolbitius bisporus* is reported as a new record for China. Detailed morphological descriptions, colour photographs and comparisons with closely-related taxa are provided.

## Introduction

The genus *Bolbitius* Fr. is well characterised by its fragile, often deliquescent basidiocarps, viscid pileus with striate margin, free lamellae, lageniform to utriform cystidia, pseudoparaphyses and a hymeniform pileipellis (Singer 1951; Watling 1982; Arnolds 2005). Molecular phylogenetics initially placed *Bolbitius* within the core lineage of *Bolbitiaceae* as a sister to *Pholiotina* (Tóth et al. 2013). This relationship was subsequently confirmed by Song and Bau (2024) through multi-locus analyses, who formally segregated the sister lineage of *Bolbitius* from *Pholiotina* sensu lato as the new genus *Conobolbitina*. A comprehensive summary of the genus, covering its nomenclatural history and ecological traits, was provided by Malysheva et al. (2015). According to Index Fungorum, the genus *Bolbitius* currently comprises 124 name records; however, some of these have since been transferred to *Conocybe*, *Conobolbitina* or other genera, leaving approximately 70 accepted species (www.indexfungorum.org, accessed 15 August 2026). It should be noted that as sister genera, *Bolbitius* and *Conobolbitina* also differ in lamellar attachment: free in most *Bolbitius* species versus adnexed in *Conobolbitina*.

Despite these advances, species delimitation within *Bolbitius* remains problematic. For instance, Malysheva et al. (2015) resolved the *B. titubans* complex into four subclades, yet the boundaries amongst these lineages remain ambiguous. Moreover, most phylogenetic studies of *Bolbitius* have relied on specimens from Europe and North America, leaving the diversity in East Asia largely unexplored. Morphology-based taxonomic treatments often fail to capture intraspecific variation and interspecific divergence within the genus, particularly for collections from undersampled regions such as China. Notably, two species, *Bolbitius roseipes* and *Bolbitius yunnanensis*, were described from Yunnan by Chiu (1973), based solely on morphology, but they lack molecular data and their phylogenetic placement remains unknown.

In this study, we examined *Bolbitius* specimens collected from northeast China in recent years. Based on morphological observations and multi-locus phylogenetic analyses (ITS, nrLSU, *tef1-α*), we identified two new species and one newly-recorded species for China. Our objectives are: (i) to formally describe the new taxa with detailed morphological illustrations; and (ii) to integrate Chinese collections into a global phylogenetic framework, compare the morphology of our materials with closely-related lineages and highlight unresolved taxonomic issues within the genus, based on our findings.

## Materials and methods

### Sampling and morphological analyses

**Figure 1.**
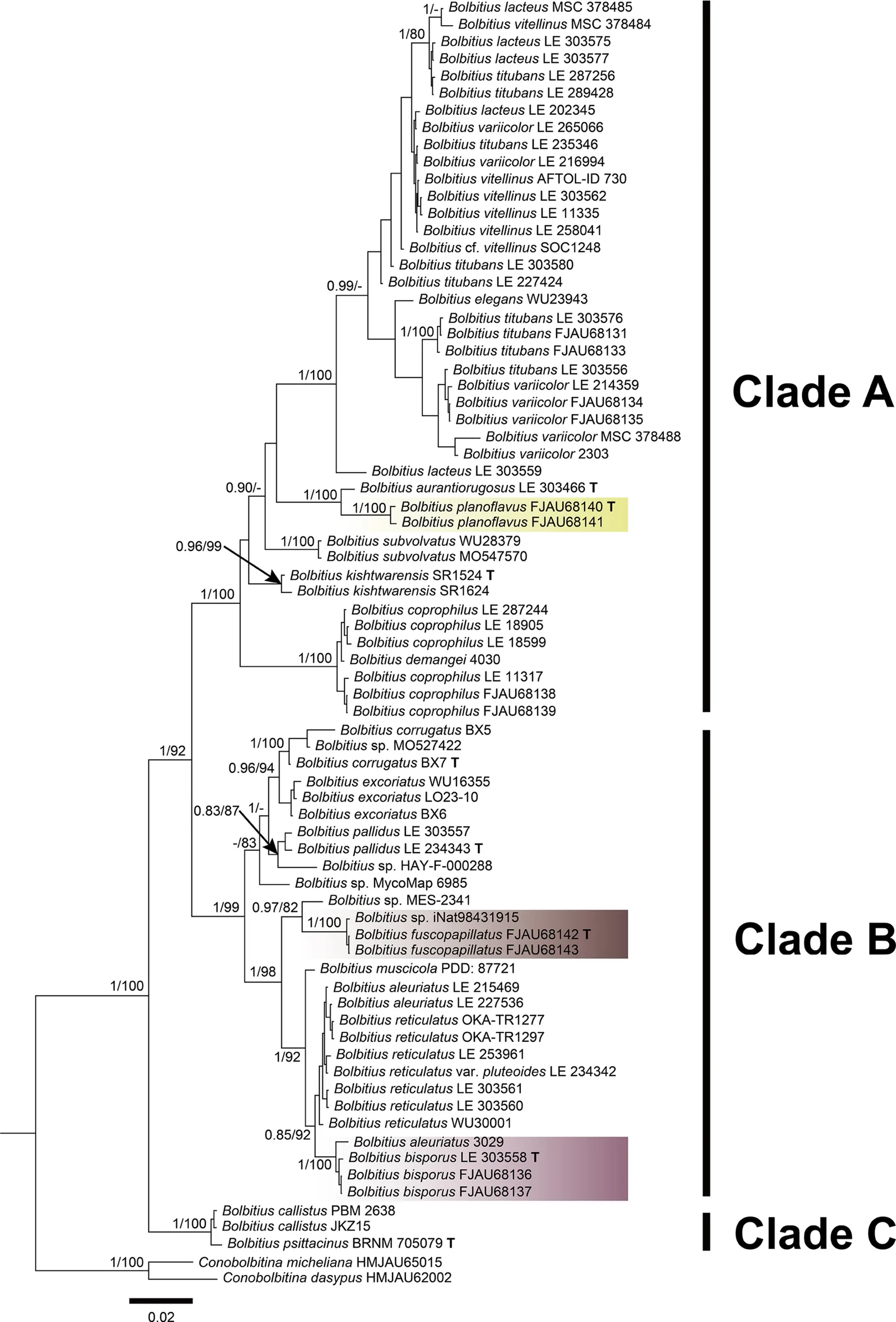
Bayesian phylogenetic tree illustrating the phylogeny of *Bolbitius*, based on combined ITS, nrLSU and *tef1-α* sequences. Support values (ML bootstrap ≥ 80%, Bayesian posterior probabilities ≥ 0.80) are given at the nodes. “T” denotes type material.

Most specimens examined in this study were collected from northeast China, with an additional collection from Yunnan Province. Field photographs (Fig. 1) documented the ecological habits, substrate, size and colour of fresh basidiomata. These fresh basidiomata were subsequently dried using a specimen dryer with hot air at 50 °C to prepare herbarium specimens, all of which are deposited in the Fungarium of Jilin Agricultural University (FJAU). Morphological descriptions were based on both fresh and dried materials, with colour designations following Kornerup and Wanscher (1978). Microscopic observations were carried out using light microscopes (Olympus CX33, Tokyo, Japan). Basidiospore measurements excluded the apiculus and are presented as “(a)b–c(d)”, where “b–c” represents the range for at least 90% of the measured values and “a” and “d” represent the extreme values. The main body (sterigmata not included) of the basidia, cheilocystidia, caulocystidia and pileipellis were measured. The notation (n/m/p) indicates that measurements were taken from “n” randomly selected basidiospores from “m” basidiomata of “p” collections. Q is the length-to-width ratio and Qm represents the mean Q ± standard deviation.

### DNA extraction, PCR amplification and sequencing

Total genomic DNA was extracted from dried specimens using a NuClean Plant Genomic DNA Kit (ComWin Biotech, CW0531M, Taizhou, China) following the manufacturer’s instructions. The primer pairs ITS1F/ITS4 (White et al. 1990; Gardes and Bruns 1993), LR0R/LR7 (Vilgalys and Hester 1990; Moncalvo et al. 2000) and EF1-983F/EF1-2218R (Rehner and Buckley 2005) were used to amplify the ITS, nrLSU and *tef1-α* regions, respectively. PCR amplification was performed on a Bio-Rad T100 Thermal Cycler in a 30 μl reaction mixture containing 2 μl of template DNA, 15 μl of 2× SanTaq PCR Master Mix (Sangon Biotech, Shanghai, China), 1.5 μl of each primer and 10 μl of ddH_2_O. The PCR programme included an annealing temperature of 53 °C for ITS and nrLSU and a touchdown annealing from 60 °C to 53 °C for *tef1-α*, each for 35 cycles. PCR products were visualised on 1% agarose gel and sent for sequencing to Sangon Biotech (Shanghai) Co., Ltd. Sequence chromatograms were checked using BioEdit v.7.0.9 (Hall 1999). The obtained sequences were deposited in GenBank.

### Phylogenetic analysis

Sequences obtained in this study and downloaded from GenBank (Table 1) were aligned, including 75 ITS, 56 nrLSU and 28 *tef1-α* sequences. The ITS, nrLSU and *tef1-α* sequences were aligned separately using the online MAFFT v.7 service with default parameters (https://mafft.cbrc.jp/alignment/server/, accessed on 11 August 2026) and subsequently refined manually in MEGA7 (Kumar et al. 2016). Subsequent trimming and phylogenetic analyses were performed in PhyloSuite v2 (Zhang et al. 2025), with ambiguously aligned regions removed using Gblocks v.0.91b (Talavera and Castresana 2007). The parameter settings were as follows: minimum number of sequences for a conserved/flank position was set to 38/38 for ITS, 29/29 for nrLSU and 15/15 for *tef1-α*, based on the number of sequences available in each respective alignment; maximum number of contiguous non-conserved positions = 8; minimum length of a block = 10; gap positions were allowed with half. The final alignment lengths were 528 bp (ITS), 885 bp (nrLSU) and 945 bp (*tef1-α*), with a total concatenated length of 2,358 bp. Missing data were coded as “?”. The best-fit partition and substitution models were selected using ModelFinder v.3.0.1 (Kalyaanamoorthy et al. 2017) under the Bayesian Information Criterion (BIC), employing the edge-unlinked partition model. The best-fit models were as follows: TIM2+F+G4 for ITS, TN+F+I for nrLSU and TN+F+G4 for *tef1-α*. Maximum Likelihood (ML) phylogenetic analyses were performed using IQ-TREE v.3.0.1 under the selected partition models, with branch support assessed, based on 1000 standard bootstrap replicates (Nguyen et al. 2015). Bayesian Inference (BI) was conducted using MrBayes v.3.2.7a (Ronquist et al. 2012) with the same three-partition scheme, but with substitution models optimised for the Bayesian framework: HKY+F+G4 for ITS, K2P+I for nrLSU, and GTR+F+G4 for *tef1-α*; all parameters were unlinked across partitions. Two independent runs (nruns = 2), each with four MCMC chains (nchains = 4, consisting of one cold and three heated chains), were performed for 2,000,000 generations, sampling trees every 1000 generations. The first 25% of sampled trees were discarded as burn-in after the average standard deviation of split frequencies fell below 0.01. The effective sample sizes (ESS) of all parameters were assessed using Tracer v.1.7.1 (Rambaut et al. 2018) to ensure adequate convergence (ESS > 200). The resulting consensus trees were visualised in FigTree v.1.4.4 and prepared for publication using Adobe Illustrator 2021.

**Table 1.** Information on the DNA sequences used to reconstruct phylogenetic trees. Sequences in bold were newly generated in this study. T = holotype.

**Taxon**	**Voucher specimen**	**GenBank accession numbers**			**Origin**	**References**
		**ITS**	**nrLSU**	***tef1-α***		
*Bolbitius aleuriatus*	LE 215469	KR425528	KR425558	–	Russia	Malysheva et al. (2015)
*Bolbitius aleuriatus*	LE 227536	KR425531	KR425561	–	Russia	Malysheva et al. (2015)
*Bolbitius aurantiorugosus*	LE 303466 T	KP033523	KR052822	–	Vietnam	Crous et al. (2015)
*Bolbitius bisporus*	LE 303558 T	KR425534	KR425564	–	Russia	Malysheva et al. (2015)
*Bolbitius bisporus*	FJAU68136	PZ502004	PZ502020	PZ512359	China	**This study**
*Bolbitius bisporus*	FJAU68137	PZ502003	PZ502019	PZ512358	China	**This study**
*Bolbitius aleuriatus*	3029	JF907770	–	–	Italy	Osmundson et al. (2013)
*Bolbitius callistus*	JKZ15	ON503052	–	–	USA	Drummond-Herdman et al. (2025)
*Bolbitius callistus*	PBM 2638	EU477860	–	–	USA	Drummond-Herdman et al. (2025)
*Bolbitius coprophilus*	LE 18905	KR425525	KR425555	–	Russia	Malysheva et al. (2015)
*Bolbitius coprophilus*	LE 11317	KR425527	KR425557	–	Russia	Malysheva et al. (2015)
*Bolbitius coprophilus*	LE 287244	KR425524	KR425554	–	Russia	Malysheva et al. (2015)
*Bolbitius coprophilus*	LE 18599	KR425526	KR425556	–	Russia	Malysheva et al. (2015)
*Bolbitius coprophilus*	FJAU68138	PZ501998	PZ502013	PZ512356	China	**This study**
*Bolbitius coprophilus*	FJAU68139	PZ501997	PZ502012	PZ512357	China	**This study**
*Bolbitius corrugatus*	BX5	PV021290	PV021296	–	Scotland	Drummond-Herdman et al. (2025)
*Bolbitius corrugatus*	BX7 T	PV364117	PV364118	–	Scotland	Drummond-Herdman et al. (2025)
*Bolbitius demangei*	4030	JF907771	–	–	Italy	Osmundson et al. (2013)
*Bolbitius elegans*	WU23943	JX968250	JX968367	–	Italy	Tóth et al. (2013)
*Bolbitius excoriatus*	WU16355	KC456418	–	–	Spain	Örstadius and Larsson (2013)
*Bolbitius excoriatus*	LO23-10	KC456419	–	–	Sweden	Örstadius and Larsson (2013)
*Bolbitius excoriatus*	BX6	PV021293	PV021295	–	Scotland	Drummond-Herdman et al. (2025)
*Bolbitius fuscopapillatus*	FJAU68142 T	PZ502006	PZ502016	PZ512350	China	**This study**
*Bolbitius fuscopapillatus*	FJAU68143	PZ502005	PZ502015	PZ512349	China	**This study**
*Bolbitius kishtwarensis*	SR1624	PV866733	–	–	India	Rajput et al. (2026)
*Bolbitius kishtwarensis*	SR1524 T	PV864814	–	–	India	Rajput et al. (2026)
*Bolbitius lacteus*	LE 202345	KR425512	KR425542	KR673349	Russia	Malysheva et al. (2015)
*Bolbitius lacteus*	LE 303577	KR425517	KR425547	KR673355	Russia	Malysheva et al. (2015)
*Bolbitius lacteus*	LE 303575	KR425516	KR425546	KR673356	Russia	Malysheva et al. (2015)
*Bolbitius lacteus*	MSC 378485	AY194520	AY293585	–	–	Drummond-Herdman et al. (2025)
*Bolbitius lacteus*	LE 303559	KR425523	KR425553	–	Russia	Malysheva et al. (2015)
*Bolbitius muscicola*	PDD: 87721	JQ694118	–	–	New Zealand	Drummond-Herdman et al. (2025)
*Bolbitius pallidus*	LE 234343 T	KR425536	KR425566	–	Russia	Malysheva et al. (2015)
*Bolbitius pallidus*	LE 303557	KR425535	KR425565	–	Russia	Malysheva et al. (2015)
*Bolbitius planoflavus*	FJAU68140 T	PZ502008	PZ502018	PZ512352	China	**This study**
*Bolbitius planoflavus*	FJAU68141	PZ502007	PZ502017	PZ512351	China	**This study**
*Bolbitius psittacinus*	BRNM 705079	EF648217	–	–	Czechia	Hausknecht et al. (2007)
*Bolbitius reticulatus*	OKA-TR1277	OK254135	OK254137	–	Turkey	Kaygusuz (2022)
*Bolbitius reticulatus*	OKA-TR1297	OK254136	OK254138	–	Turkey	Kaygusuz (2022)
*Bolbitius reticulatus*	LE 253961	KR425529	KR425559	–	Russia	Malysheva et al. (2015)
*Bolbitius reticulatus*	LE 303560	KR425533	KR425563	–	Russia	Malysheva et al. (2015)
*Bolbitius reticulatus*	LE 303561	KR425530	KR425560	–	Russia	Malysheva et al. (2015)
*Bolbitius reticulatus*	WU30001	JX968249	JX968366	–	Hungary	Tóth et al. (2013)
Bolbitius reticulatus var. pluteoides	LE 234342	KR425532	KR425562	–	Russia	Malysheva et al. (2015)
*Bolbitius subvolvatus*	WU28379	JX968248	JX968365	–	Italy	Tóth et al. (2013)
*Bolbitius subvolvatus*	MO547570	PQ137777	–	–	USA	Rajput et al. (2026)
*Bolbitius titubans*	LE 303580	KR425537	–	–	Russia	Malysheva et al. (2015)
*Bolbitius titubans*	LE 303556	KR425522	KR425552	KR673347	Russia	Malysheva et al. (2015)
*Bolbitius titubans*	LE 289428	KR425519	KR425549	KR673351	Russia	Malysheva et al. (2015)
*Bolbitius titubans*	LE 235346	KR425509	KR425539	KR673353	Russia	Malysheva et al. (2015)
*Bolbitius titubans*	LE 287256	KR425518	KR425548	KR673354	Russia	Malysheva et al. (2015)
*Bolbitius titubans*	LE 227424	KR425510	KR425540	–	Russia	Malysheva et al. (2015)
*Bolbitius titubans*	LE 303576	KR425520	KR425550	KR673357	Russia	Malysheva et al. (2015)
*Bolbitius titubans*	FJAU68131	PZ501999	PZ502014	PZ512355	China	**This study**
*Bolbitius titubans*	FJAU68133	PZ502002	PZ502023	PZ512360	China	**This study**
*Bolbitius variicolor*	LE 216994	KR425508	KR425538	KR673346	Russia	Malysheva et al. (2015)
*Bolbitius variicolor*	LE 265066	KR425511	KR425541	KR673348	Russia	Malysheva et al. (2015)
*Bolbitius variicolor*	LE 214359	KR425521	KR425551	KR673358	Sweden	Malysheva et al. (2015)
*Bolbitius variicolor*	MSC 378488	AY194535	AY293586	–	–	Drummond-Herdman et al. (2025)
*Bolbitius variicolor*	2303	JF907768	–	–	Italy	Osmundson et al. (2013)
*Bolbitius variicolor*	FJAU68134	PZ502001	PZ502021	PZ512353	China	**This study**
*Bolbitius variicolor*	FJAU68135	PZ502000	PZ502022	PZ512354	China	**This study**
*Bolbitius vitellinus*	LE 303562	KR425513	KR425543	KR673350	Russia	Malysheva et al. (2015)
*Bolbitius vitellinus*	LE 258041	KR425514	KR425544	KR673352	Russia	Malysheva et al. (2015)
*Bolbitius vitellinus*	LE 11335	KR425515	KR425545	–	Russia	Malysheva et al. (2015)
*Bolbitius vitellinus*	MSC 378484	AY194519	AY293583	–	USA	Drummond-Herdman et al. (2025)
*Bolbitius vitellinus*	AFTOL-ID 730	DQ200920	AY691807	DQ408148	USA	Drummond-Herdman et al. (2025)
*Bolbitius cf. vitellinus*	SOC1248	FJ235157	–	–	USA	Drummond-Herdman et al. (2025)
*Bolbitius* sp.	MycoMap 6985	MK564538	–	–	USA	Drummond-Herdman et al. (2025)
*Bolbitius* sp.	HAY-F-000288	OR387405	–	–	USA	Drummond-Herdman et al. (2025)
*Bolbitius* sp.	MO527422	PP047665	–	–	USA	Drummond-Herdman et al. (2025)
*Bolbitius* sp.	iNat98431915	OP549060	–	–	USA	Drummond-Herdman et al. (2025)
*Bolbitius* sp.	MES-2341	MH930330	–	–	Chile	Drummond-Herdman et al. (2025)
*Conobolbitina micheliana*	HMJAU65015	OR995677	OR994080	PP000869	China	Song and Bau (2024)
*Conobolbitina dasypus*	HMJAU62002	OR995661	OR995711	PP000854	China	Song and Bau (2024)

## Results

### Phylogenetic analysis

The final concatenated dataset comprised 75 taxa and 2,358 bp (total aligned length) from ITS, nrLSU and *tef1-α*. *Conobolbitina micheliana* (HMJAU65015) and *C. dasypus* (HMJAU62002) were designated as the outgroup. Based on the phylogenetic analyses, the genus *Bolbitius* is divided into three main clades (A, B and C), as shown in the Bayesian tree (Fig. 1). The ML tree (Suppl. material 1: fig. S2) was topologically congruent with the Bayesian tree, recovering the same three clades. In Fig. 1, nodal support is indicated only for nodes with PP ≥ 0.80 and/or MLBS ≥ 80%; more detailed support values are available on the ML tree in the Suppl. material 1.

Phylogenetic analyses revealed that the *Bolbitius* specimens obtained in this study fell into two major clades, designated as A and B. In clade A, *B. planoflavus* formed a well-supported sister clade with the *B. aurantiorugosus* holotype (LE 303466; PP = 1.0, MLBS = 100%), though this relationship is based on ITS and nrLSU as *tef1-α* is missing for that holotype. Within clade B, both the new species *B. fuscopapillatus* and the newly-recorded *B. bisporus* were recovered. *B. fuscopapillatus* clustered with unidentified sequences *Bolbitius* sp. iNat98431915 (GenBank OP549060; strong support: PP = 1.0, MLBS = 100%) and *Bolbitius* sp. MES-2341 (GenBank MH930330; moderate support: PP = 0.97, MLBS = 82%), but, since both lack nrLSU and *tef1-α*, support relies mainly on ITS. Two of our specimens (FJAU68136, FJAU68137) grouped with the *B. bisporus* holotype (LE 303558; PP = 1.0, MLBS = 100%), but as that holotype lacks *tef1-α*, this relationship is mainly supported by ITS and nrLSU, while our two specimens (possessing all three loci) remained consistently placed in the concatenated analysis.

To evaluate whether the phylogenetic conclusions were influenced by the uneven availability of locus data across taxa, we performed additional analyses using ITS-only and ITS+nrLSU datasets (Suppl. material 1: figs S3, S4). The resulting topologies were largely congruent with the concatenated three-locus tree, with stable placements of *B. fuscopapillatus* and *B. bisporus*, indicating that the key relationships are robust despite the absence of *tef1-α* data for several critical reference sequences.

### Taxonomy

#### Bolbitius planoflavus

Taxon classificationFungiAgaricalesBolbitiaceae

T. Bau & H. Cheng sp. nov.

299A17DB-73D6-5272-A85C-0C34DD93C73F

864018

Figs 2k, l, 3

##### Etymology.

“planoflavus” refers to the species’ pileus being both plane and pale yellow when mature.

**Figure 2.**
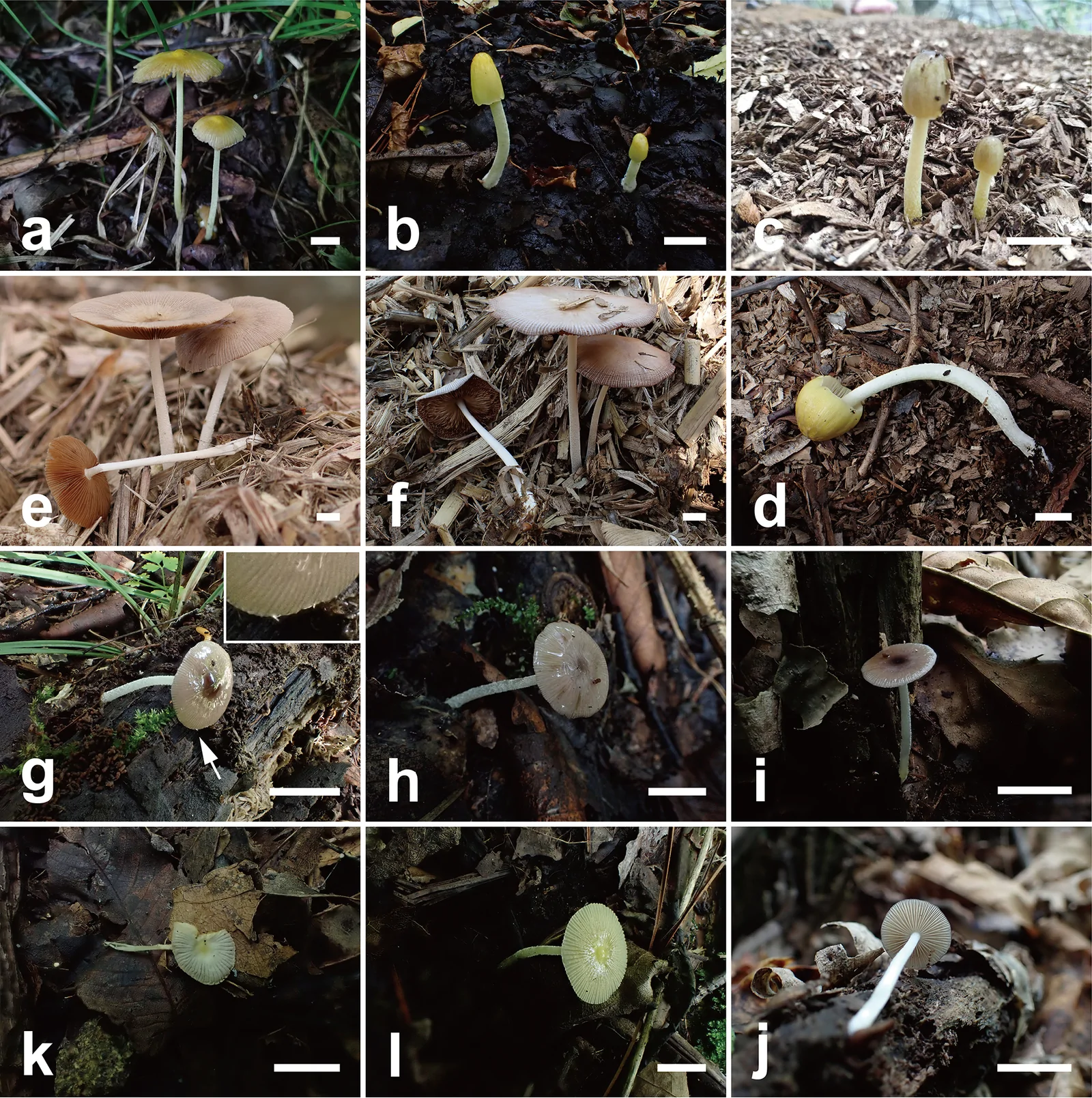
Basidiomata of *Bolbitius* species. **a, b**. *B. titubans* (FJAU68133, FJAU68132); **c, d**. *B. variicolor* (FJAU68135, FJAU68134); **e, f**. *B. coprophilus* (FJAU68139, FJAU68138); **g, h**. *B. fuscopapillatus* (FJAU68142 T, FJAU68143); **i, j**. *B. bisporus* (FJAU68137); **k, l**. *B. planoflavus* (FJAU68141, FJAU68140 T); T = holotype. White arrows in (**g**) indicate veil remnants, with an enlarged view shown in the upper right corner. Scale bars: 1 cm.

**Figure 3.**
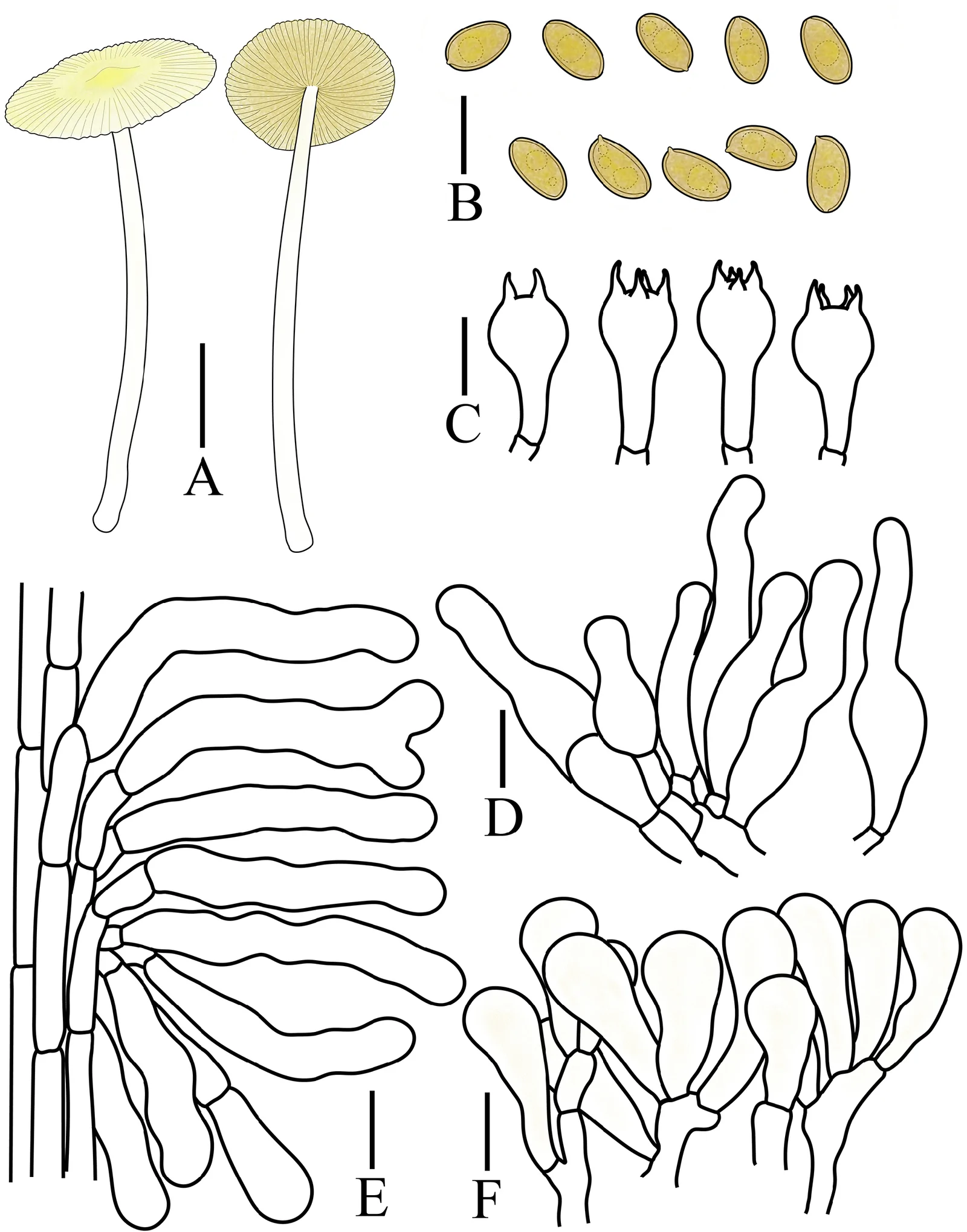
*Bolbitius planoflavus* (FJAU68140). **A**. Basidiomata; **B**. Basidiospores; **C**. Basidia; **D**. Cheilocystidia; **E**. Stipitipellis and caulocystidia; **F**. Pileipellis elements. Scale bars: 1 cm (**A**); 10 μm (**B**–**F**).

##### Holotypus.

China • Jilin Province, Dunhua City, Laobai Mountain, 21 Jul 2025, 44°07'31"N, 127°59'16"E, alt. 273 m, Hong Cheng, C2572105 (FJAU68140).

##### Diagnosis.

*Bolbitius planoflavus* is characterised by its distinctly applanate, pale yellow pileus when mature; ellipsoid to oblong basidiospores (9.0–10.5 μm × 5.2–5.8 μm) with a distinct germ pore; occasional 2-spored basidia; and absence of clamp-connections. It differs from other yellow-pileate species as follows: from *B. lacteus* (whitish pileus, larger spores, deliquescent gills); from *B. aurantiorugosus* (reddish-orange pileus tints, larger boletoid spores); and from *B. titubans* (bright yellow, non-applanate pileus).

##### Description.

***Basidiomata*** small-sized, pileus 1.5–2 cm in diameter, applanate when mature, slightly umbonate or slightly depressed at centre; pastel yellow to pale yellow (2A4 to 2A3) at the centre gradually paler towards the margin to pale yellow (2A3) or yellowish-white (2A2). Pileus viscid, especially at the centre, margin sulcate-striate up to 2/3 of the radius. Context thin, with no specific odour. Lamellae free, moderately crowded, unequal in length, ventricose, white when young, gradually becoming orange-white (5A2) to light orange (5A3) with age, with the edges nearly white in dried specimens. Stipe 3–5 cm long, 2–4 mm thick, cylindrical, fistulose, fragile, white (1A1) to yellowish-white (1A2), longitudinally striate and covered with white powdery to flocculose pubescence. Stipe base slightly enlarged.

***Basidiospores*** (60/2/2) 9.0–10.5(–12.2) μm × (5.0–)5.2–5.8(–6.3) μm, Q = (1.51–)1.58–2.05(–2.25), Qm = 1.81(± 0.17), ellipsoid to oblong in frontal view, similar in side view, but with a slight suprahilar depression, thick-walled, smooth, with distinct germ pore, golden yellow (5B7) to brownish-yellow (5C7) in potassium hydroxide (KOH) solution. Basidia 18–24 × 9.5–11 μm, broadly clavate, 4-spored, occasionally 2-spored, sterigmata 3–5 μm long, with vacuolar contents. Cheilocystidia (24)27–47(–52) × 7–10(–12) μm, clavate to narrow utriform, sometimes with a long neck and rounded apex, hyaline, thin-walled. Pleurocystidia absent. Stipitipellis a cutis. Caulocystidia (20–)24–47(–50) × 7–8 μm, cylindrical to narrowly clavate, often flexuose, constricted near the base, rounded at apex, occasionally furcate, hyaline, thin-walled. Pileocystidia absent. Pileipellis hymeniform, composed of clavate to broadly clavate elements, (15–)18–26 × 7–10(–12) μm, thin walled, nearly colourless. Clamp-connections were not observed in any of the examined hyphae, including those from the pileus, lamellae and stipe.

##### Habitat.

Solitary, on rotten leaves or deeply decayed wood in broad-leaved or mixed forests dominated by *Quercus* species, with *Pinus* as associated trees; usually in humid and shaded microhabitats; fruiting in summer.

##### Known distribution.

Currently known only from Jilin Province, China.

##### Additional specimens examined.

China • Jilin Province, Ji’an City, Wunüfeng National Forest Park, 25 Jul 2025, 41°16'22"N, 126°07'12"E, alt. 330 m, Hong Cheng, C2572519 (FJAU68141).

##### Notes.

Within the genus *Bolbitius*, only a few species are known to have a distinctly yellow pileus, including *Bolbitius corrugatus* and the *Bolbitius titubans* species complex. *Bolbitius planoflavus* is distinguished from these by its distinctly applanate (flattened) pileus when mature, whereas other yellow-capped species typically retain a convex or plano-convex shape. From *Bolbitius corrugatus* (Drummond-Herdman et al. 2025), *B. planoflavus* further differs in basidiospore morphology (ellipsoid to oblong in frontal view vs. irregular in *B. corrugatus*) and ecology (on rotten leaves or deeply decayed wood in broad-leaved or mixed forests vs. on old cow dung in upland grassland). Additionally, compared to the *Bolbitius titubans* complex as described by Malysheva et al. (2015), *B. planoflavus* possesses notably smaller basidiospores. *Bolbitius flavus* (Murrill) Sacc. & Trott. also exhibits a distinctly applanate pileus at maturity with a colour similar to that of *B. planoflavus*. However, *B. flavus* is generally larger (pileus 4–7 cm in wide; stipe 6–10 cm in long), grows caespitosely on horse dung (whereas *B. planoflavus* is saprotrophic on rotten leaves or deeply decayed wood in forests) and possesses markedly larger basidiospores (14 μm × 8 μm) compared to the notably smaller spores of *B. planoflavus*.

According to molecular data (ITS sequences), *B. planoflavus* shows 95.24% sequence identity with its closest relative *B. aurantiorugosus* (holotype LE 303466, GenBank KP033523) and 89.47% identity with *B. titubans* (LE 303580, KR425537).

#### Bolbitius fuscopapillatus

Taxon classificationFungiAgaricalesBolbitiaceae

T. Bau & H. Cheng sp. nov.

7F5AE3ED-68AC-52A3-8D97-A94A701A2592

864019

Figs 2g, h, 4

##### Etymology.

From Latin *fuscus* (violet-brown) and *papillatus* (with a small papilla), referring to the violet-brown papillate umbo of the pileus.

**Figure 4.**
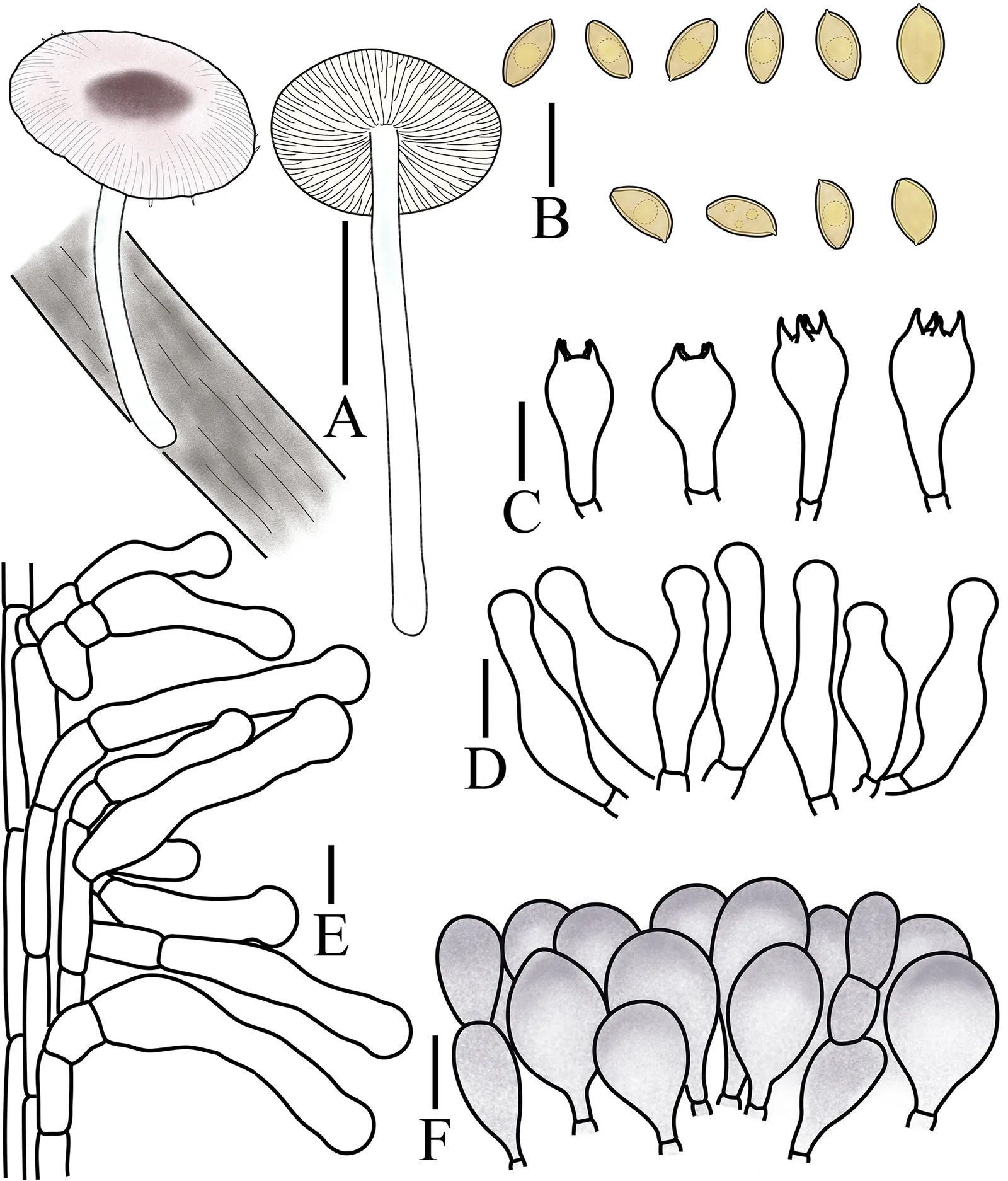
*Bolbitius fuscopapillatus* (FJAU68142). **A**. Basidiomata; **B**. Basidiospores; **C**. Basidia; **D**. Cheilocystidia; **E**. Stipitipellis and caulocystidia; **F**. Pileipellis elements. Scale bars: 1 cm (**A**); 10 μm (**B**–**F**).

##### Holotypus.

China • Heilongjiang Province, Hailun City, Chenjiadian Forest Farm, 12 Aug 2025, 47°27'36"N, 127°04'48"E, alt. 210 m, Hong Cheng, C2581212 (FJAU68142).

##### Diagnosis.

*Bolbitius fuscopapillatus* is characterised by its violet-brown papillate umbo, a purplish-white margin bearing faint veil remnants, consistently 4-spored basidia, ellipsoid to oblong basidiospores measuring 8.0–9.5 μm × 4.3–5.0 μm and the absence of clamp-connections. It is distinguished from *B. bisporus* by its 4-spored (vs. predominantly 2-spored) basidia and from *B. reticulatus* by its dark brown pileus centre (vs. distinctly paler pileus, ranging from white-pink or greyish-beige to white-grey), smaller basidiospores [8.0–9.5 × 4.3–5.0 μm vs. (8.5–)9.6–11.8(–12.7) μm × (5.0–)5.5–6.5(–7.2) μm] and lacking clamp-connections (vs. present).

##### Description.

***Basidiomata*** small-sized, pileus 1–1.8 cm in diameter, convex to plano-convex when mature, slightly umbonate; violet brown (10F8, 10E8) at the centre, the umbo notably darker, margin purplish-white (14A2). Pileus surface very viscid, with radiating sulcate-striate; margin with faint whitish (1A1) veil remnants, evanescent. Context thin, with no specific odour. Lamellae free, moderately crowded, unequal in length, ventricose, white when young, gradually becoming pale yellow (4A3) to greyish-yellow (4B4), with nearly white edges in dried specimens. Stipe 3–4 cm long, 2–3 mm thick, cylindrical, fistulose, fragile, white (1A1), longitudinally striate and covered with white powdery to flocculose pubescence. Stipe base slightly enlarged.

***Basidiospores*** (60/2/2) (7.3–) 8.0–9.5 μm × 4.3–5.0 μm, Q = (1.62–)1.66–2.01(–2.10), Qm = 1.85(± 0.11), ellipsoid to oblong in frontal view, similar in side view, but with a slight suprahilar depression, thick-walled, smooth, with distinct germ pore, golden yellow (3B5) to brownish-yellow (3C7) in KOH solution. Basidia 16–21 μm × 7.8–10 μm, broadly clavate, 4-spored, sterigmata 3–5 μm long, with vacuolar contents. Cheilocystidia (18–)24–35 μm × (5.5–)6.6–11 μm, clavate to narrowly utriform, with a rounded apex, hyaline, thin-walled. Pleurocystidia absent. Stipitipellis a cutis. Caulocystidia (26–)33–52(–60) μm × 7.5–11.5 μm, cylindrical to narrowly clavate, narrowly utriform, with rounded apex or slightly capitate, hyaline, thin-walled. Pileocystidia absent. Pileipellis hymeniderm, composed of a mixture of thin-walled elements bearing dull violet (14D4, 14E4) pigment: mostly subglobose to pyriform elements (11–24 μm × 10–21 μm), together with fewer clavate to broadly clavate elements (11–27 μm × 6.5–7.5 μm). Clamp-connections were not observed in any of the examined hyphae, including those from the pileus, lamellae and stipe.

##### Habitat.

Solitary, on rotten wood in broad-leaved forests dominated by *Quercus* species, with *Betula* as associated trees; usually in humid and shaded microhabitats; fruiting in autumn.

##### Known distribution.

Currently known only from Heilongjiang Province, China.

##### Additional specimens examined.

China • Heilongjiang Province, Hailun City, Chenjiadian Forest Farm, 25 Aug 2025, 47°27'42"N, 127°04'55"E, alt. 209 m, Hong Cheng, C2581214 (FJAU68143).

##### Notes.

*Bolbitius fuscopapillatus* is morphologically similar to *B. bisporus* due to their sharing wood-inhabiting habitat and whitish veil remnants on the pileus margin. However, *B. bisporus* is characterised by predominantly 2-spored basidia, whereas *B. fuscopapillatus* consistently has 4-spored basidia. Our specimen FJAU68143 also exhibits a certain macro-morphological resemblance to *Bolbitius reticulatus* (OKA-TR1277). Nevertheless, the two can be readily distinguished by several key features. In *B. fuscopapillatus*, the centre of the pileus is dark brown, while in *B. reticulatus*, the entire pileus is distinctly paler, ranging from white-pink or greyish-beige to white-grey. Moreover, *B. reticulatus* produces significantly larger basidiospores, measuring (8.5–)9.6–11.8(–12.7) μm × (5.0–)5.5–6.5(–7.2) μm, compared to 8.0–9.5 μm × 4.3–5.0 μm in *B. fuscopapillatus*. Additionally, clamp-connections are present in *B. reticulatus*, but are entirely absent in *B. fuscopapillatus*. *B. fuscopapillatus* also resembles *B. malesianus* Watling, a species described from Sabah, Malaysian Borneo (Watling 1994). Although both share a purplish pileus, wood-inhabiting habit, and 4-spored basidia, *B. malesianus* differs by its larger pileus (25–37 mm vs. 10–18 mm) and more elongate basidiospores [8.7–9.6(–11) μm × 3.9–4.8(–5.2) μm vs. (7.3–)8.0–9.5 μm × 4.3–5.0 μm]. Additionally, *B. malesianus* has a rimulose pileus margin (vs. sulcate-striate with whitish veil remnants in *B. fuscopapillatus*). The two species also have disjunct distributions (Borneo vs. north-eastern China). Therefore, despite the absence of molecular data for *B. malesianus*, the combined morphological and biogeographical evidence supports treating *B. fuscopapillatus* as a distinct species.

Based on ITS data, *B. fuscopapillatus* shares 99.72% similarity with an unnamed *Bolbitius* sp. (OP549060), differing by two nucleotides (one A deletion and one T substitution). Due to the absence of morphological data for *Bolbitius* sp. (OP549060), we tentatively regard it as a closely-related collection that may represent the same species, but it is not treated as a confirmed distribution record. By contrast, *B. fuscopapillatus* differs from *B. muscicola* (JQ694118) by 6.24% ITS divergence; this genetic distance, combined with the morphological evidence presented above, supports their treatment as separate species.

#### Bolbitius bisporus

Taxon classificationFungiAgaricalesBolbitiaceae

E.F. Malysheva

622EFF5E-0F9D-5442-9734-41323F8A0418

Figs 2i, j, 5

##### Description.

***Basidiomata*** small-sized, pileus 0.8–1.8 cm in diameter, convex to applanate when mature; greyish-violet (18D4) at the centre, transitioning to reddish-brown (9D4), with the margin grey (18B1) to white (18A1). Pileus surface viscid, with radiating sulcate-striae; margin with faint whitish (1A1) veil remnants, evanescent. Context thin, with no specific odour. Lamellae free, moderately crowded, unequal in length, ventricose, white when young, gradually becoming pale yellow (4A3), with the edges nearly white in dried specimens. Stipe 2–4 cm long, 2–3 mm thick, cylindrical, fistulose, fragile, white (1A1), longitudinally striate and covered with white powdery to flocculose pubescence. Stipe base slightly enlarged, with surface sometimes pale yellow (2A3).

**Figure 5.**
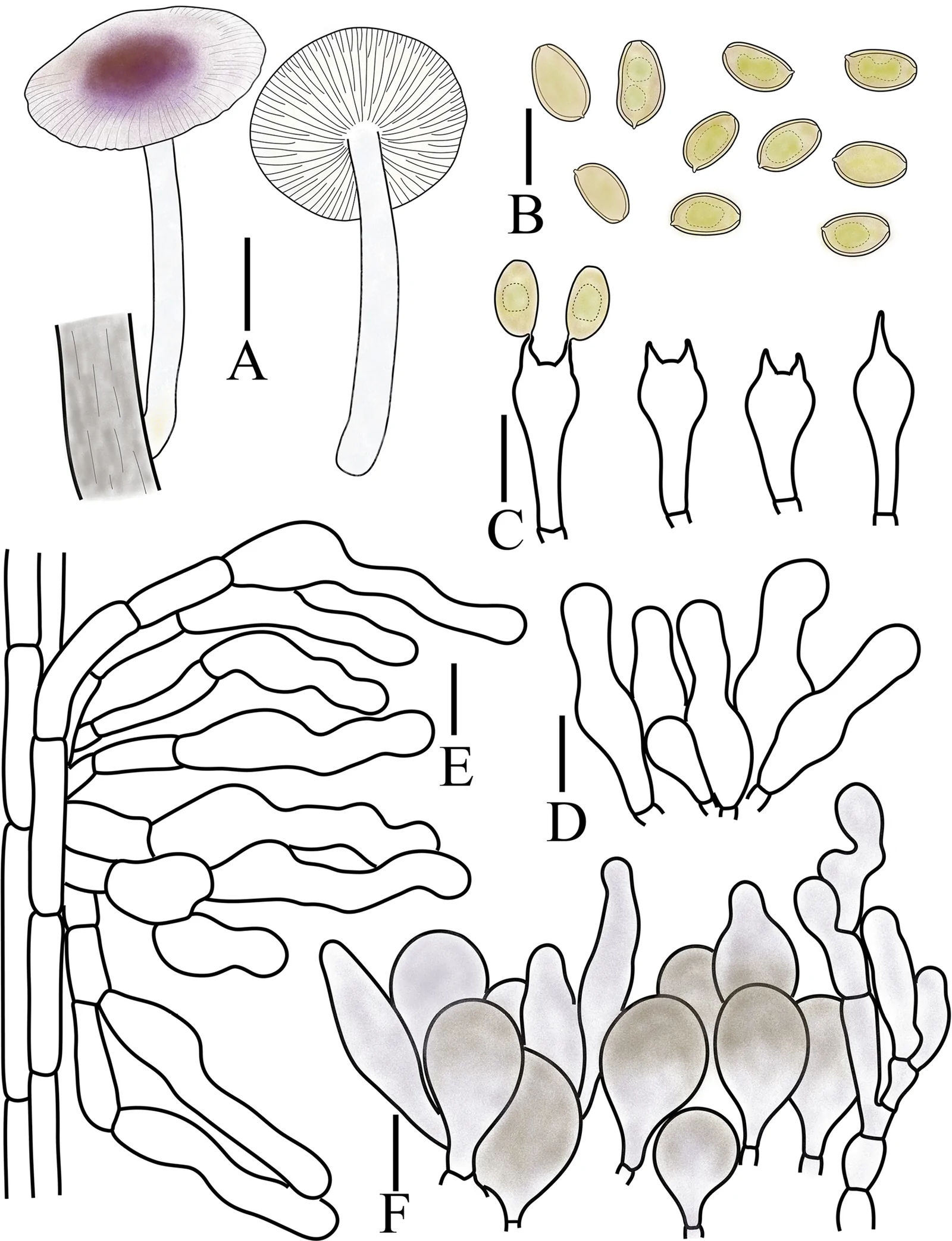
*Bolbitius bisporus* (FJAU68137). **A**. Basidiomata; **B**. Basidiospores; **C**. Basidia; **D**. Cheilocystidia; **E**. Stipitipellis and caulocystidia; **F**. Pileipellis elements. Scale bars: 0.5 cm (**A**); 10 μm (**B**–**F**).

***Basidiospores*** (60/2/2) 9.8–12.7 μm × 5.1–6.8 μm, Q = 1.63–1.97, Qm = 1.79 (± 0.09), ellipsoid to oblong in frontal view, similar in side view, but slightly flattened or with a slight suprahilar depression, thick-walled, smooth, with distinct germ pore, greyish-yellow (4B5) to greyish-orange (5B5) in KOH solution. Basidia 16–2 μm 4 × 6.5–8 μm, broadly clavate, predominantly 2-spored, occasionally 1-spored, sterigmata 3–5 μm long, with vacuolar contents. Cheilocystidia 11–32 μm × 6.0–7.8 μm, clavate to narrowly utriform, with a rounded apex, hyaline, thin-walled. Pleurocystidia absent. Stipitipellis a cutis. Caulocystidia (26–)33–51 μm × 9–13 μm, cylindrical to narrowly clavate or narrowly utriform, neck flexuous, apex rounded, hyaline, thin-walled. Pileocystidia absent. Pileipellis a hymeniderm, composed of a mixture of thin-walled elements containing greyish-magenta (13D3) to purplish-grey (13D2) or light brown (7D4) pigment: mostly subglobose to pyriform elements (17–41 μm × 10–20 μm), together with fewer clavate to narrowly cylindrical elements (18–32 μm × 4.2–6.2 μm). Clamp-connections were not observed in any of the examined hyphae, including those from the pileus, lamellae and stipe.

##### Habitat.

Solitary, on rotten wood in broad-leaved forests dominated by Quercus species, with Betula as associated trees; usually in humid and shaded microhabitats; fruiting in autumn.

##### Known distribution.

Russia (type locality, Malysheva et al. (2015)); China: Jilin Province (this study, new record).

##### Additional specimens examined.

China • Jilin Province, Ji’an City, Wunüfeng National Forest Park, 41°16'22"N, 126°07'12"E, alt. 954 m, 7 Jul 2023, Qian-Ru Liu, R23728 (FJAU68137); • Tonghua City, Baijifeng National Forest Park, 41°33'56.43"N, 126°05'14.52"E, alt. 836 m, 7 Jul 2023, Qian-Ru Liu, R23740 (FJAU68136).

##### Notes.

Although specimens from this study exhibit some morphological variation from the holotype (LE 303558)—most notably in having smaller spores (9.8–12.7 μm × 5.1–6.8 μm vs. 13–17 μm × 6.5–8.5 μm in the type)—the key diagnostic character, the predominantly bisporic basidia, is retained. Furthermore, the pileipellis elements and their arrangement, as well as the solitary habit, are also consistent with the type material. Additionally and importantly, the ITS sequence of our collections shows 99.85% similarity with the *B. bisporus* holotype (LE 303558), differing by only a single nucleotide. This high level of sequence identity strongly supports conspecificity, as it falls well within the range of intraspecific variation commonly observed in fungi. We therefore interpret the spore size difference as intraspecific variation and confidently identify our Chinese collections as *B. bisporus*, representing a new record from China.

## Discussion

In summary, this study describes two new species, *Bolbitius planoflavus* and *Bolbitius fuscopapillatus* and reports one newly-recorded species, *B. bisporus*, for China. The taxonomic conclusions are well supported by combined morphological and molecular phylogenetic evidence. Phylogenetically, *B. planoflavus* was recovered within Clade A, while *B. fuscopapillatus* and *B. bisporus* were placed in Clade B. Notably, this phylogenetic distribution shows a certain correlation with pileipellis morphology: Clade A species examined in this study (e.g. *B. planoflavus*) exhibit a relatively homogeneous hymeniform pileipellis composed mainly of clavate, hyaline to pale yellowish cells without dark pigments, whereas certain Clade B species, including *B. fuscopapillatus* and *B. bisporus*, display a hymeniform pileipellis comprising two cell types (subglobose and clavate) with dark purple intracellular pigments. Nevertheless, this trend is observed amongst a limited number of taxa within Clades A and B (Suppl. material 1: fig. S1) and its generality requires further evaluation with broader sampling.

Despite these contributions, a well-established, molecularly verified infrageneric classification framework for *Bolbitius* remains lacking. Contu (2010) proposed two sections within the genus, based on morphological traits, but the original diagnoses do not provide fully satisfactory delimitations for these sections. In our materials, *B. fuscopapillatus* and *B. bisporus* exhibit faint whitish veil remnants on the pileus margin, whereas *B. reticulatus* (OKA-TR1277), which also falls within Clade B, appears to lack veil remnants, but possesses clamp-connections (Kaygusuz 2022). Clamp-connections were not observed in any of our specimens. Given the limited information available in the original diagnoses and the conflicting distribution of these morphological characters within a well-supported clade, we refrain from assigning our taxa to any sectional classification. A broader taxon sampling with both molecular and morphological data will be required to further evaluate the utility of clamp-connections and veil remnants as phylogenetically informative characters at the sectional level in *Bolbitius*.

Collectively, our findings highlight the need for broader taxon sampling and additional molecular data to resolve the infrageneric phylogeny of *Bolbitius*. We recognise that each of the new taxa is represented by only two collections. Although the available specimens consistently support the morphological and molecular delimitation of these taxa, future collections from additional localities will be essential to reveal the full range of morphological variation within each species and to clarify species boundaries. Furthermore, given that our study adds two new species and one newly-recorded species from China, it is plausible that Asia harbours a potentially higher diversity of *Bolbitius* than currently recognised. Future studies should integrate more extensive collections, particularly from under-sampled regions in Asia and employ phylogenomic approaches to establish a stable, character-based classification framework for this taxonomically challenging genus. Continued efforts to accumulate both more specimens per species and a broader representation of taxa will be critical for achieving a robust infrageneric classification.

## Supplementary Material

XML Treatment for Bolbitius planoflavus

XML Treatment for Bolbitius fuscopapillatus

XML Treatment for Bolbitius bisporus

## Additional information

### Artificial Intelligence (AI) use

The authors accept full responsibility for the content of the manuscript, including the disclosure of any use of AI.

No AI tools were used in the preparation of this manuscript.

### Author ORCIDs

H. Cheng https://orcid.org/0009-0003-0856-1048

T. Bau https://orcid.org/0000-0003-2461-9345

### Data availability

All of the data that support the findings of this study are available in this article. The supplementary materials, including the phylogenetic alignment files and Suppl. material 1: figs S1–S4, have been deposited in Zenodo (https://doi.org/10.5281/zenodo.20576333).

## Supplementary materials

Supplementary material 1 Supplementary figures Data type docx https://binary.pensoft.net/file/1788119This dataset is made available under the Open Database License (http://opendatacommons.org/licenses/odbl/1.0/). The Open Database License (ODbL) is a license agreement intended to allow users to freely share, modify, and use this Dataset while maintaining this same freedom for others, provided that the original source and author(s) are credited. Hong Cheng, Tolgor Bau
