# Supplementary material for: Two new species and one new record of Bolbitius (Bolbitiaceae, Agaricales) from northeast China
Source: MycoKeys. 2026 Sep 23;140:299–316. doi: 10.3897/mycokeys.140.203173 (PMC13628148; doi:10.3897/mycokeys.140.203173)
Supplement: Supplementary file 1 [file mycokeys-140-299-s001.docx]

**Supplementary Figure S1.** A1–A4, *Bolbitius fuscopapillatus* (FJAU68142); B1–B4, *B. bisporus* (FJAU68137); C1–C4, *B. coprophilus* (FJAU68139); D1–D4, *B. planoflavus* (FJAU68140); E1–E4, *B. variicolor* (FJAU68135); F1–F4, *B. titubans* (FJAU68133). A1–F1: pileipellis elements; A2–F2: basidiospores; A3–F3: cheilocystidia; A4–F4: stipitipellis and caulocystidia.

**Supplementary Figure S2.** Maximum likelihood (ML) tree illustrating the phylogeny of *Bolbitius* based on combined ITS, nrLSU, and *tef1-α* sequences. Support values (ML bootstrap ≥ 60%, Bayesian posterior probabilities ≥ 0.60) are given at the nodes. “T” denotes type material.

**Supplementary Figure S3.** Bayesian phylogenetic tree of *Bolbitius* inferred from ITS sequence data. Bayesian posterior probabilities (≥0.60) and bootstrap support values (≥60%) are shown at the nodes. “T” denotes type material.

**Supplementary Figure S4.** Bayesian phylogenetic tree of *Bolbitius* inferred from a concatenated dataset of ITS and nrLSU sequences. Bayesian posterior probabilities (≥0.60) and bootstrap support values (≥60%) are shown at the nodes. “T” denotes type material.
